# Vascular, inflammatory and immune factors associated with acute ischemic events in Takayasu arteritis: a 10-year retrospective matched case-control study

**DOI:** 10.3389/fimmu.2026.1866057

**Published:** 2026-09-11

**Authors:** Yang Liu, Ying Liu, Haizhuan An, Qian Li, Sumiao Liu, Ke Xu

**Affiliations:** Third Hospital of Shanxi Medical University, Shanxi Bethune Hospital, Shanxi Academy of Medical Sciences, Tongji Shanxi Hospital, Taiyuan, China

**Keywords:** acute ischemic events, IgA, IL-6, inflammation, Takayasu arteritis, Th cell

## Abstract

**Objectives:**

Acute ischemic events (AIE) constitute life-threatening complications of Takayasu arteritis (TAK). This study aimed to identify vascular, inflammatory and immune factors associated with concurrent AIE in TAK patients.

**Methods:**

We conducted a 10-year retrospective 1:2 matched case-control study including TAK patients without exposure to glucocorticoids, immunosuppressants, or antithrombotic agents for at least 3 months. Clinical characteristics, disease activity, inflammatory markers, and lymphocyte subsets were compared between patients with and without AIE. Three sequential multivariable logistic regression models were constructed to identify variables independently associated with AIE. Model discrimination was evaluated using receiver operating characteristic (ROC) analysis with 1000-bootstrap internal validation, while calibration and decision curve analysis (DCA) were performed as exploratory within-sample assessments.

**Results:**

Thirty-seven patients with AIE and 74 matched TAK control without AIE were included. The mean age at TAK onset in the AIE group was 34.92 ± 10.83 years (range: 16–58 years). Conventional markers including ESR, CRP and homocysteine showed no independent correlation with AIE. Patients with AIE presented substantially higher frequencies of coronary, anterior cerebral, middle cerebral, and femoral artery stenosis, alongside significantly elevated ITAS-2010 scores, serum IgA and IL-6 levels, and peripheral Th cell counts (all *p* < 0.05). Severe long-term functional sequelae were also more frequent in the AIE group. Multivariate analysis confirmed that stenosis at these four arterial sites, together with elevated ITAS-2010 score, IgA, IL-6, and Th cell counts, were independently associated with AIE. The fully integrated vascular-inflammation-immunity model yielded an apparent AUC of 0.942 (95%CI 0.898-0.985) and a bootstrap optimism-corrected AUC of 0.908 (95%CI 0.843-0.959), with a corrected Brier score of 0.1191. Calibration and DCA supported satisfactory within-sample model performance but should be interpreted as exploratory because of the matched case-control design.

**Conclusions:**

Specific vascular stenosis, high disease activity, elevated serum IgA and IL-6 levels, and increased peripheral Th cell counts are independently associated with AIE in TAK. These findings provide additional insight into the vascular, inflammatory, and immune characteristics accompanying ischemic complications in TAK and warrant confirmation in prospective multicenter studies.

## Introduction

Takayasu arteritis (TAK) is a rare, chronic granulomatous vasculitis predominantly affecting the aorta and its major branches, with a marked predilection for women under 40 years of age (1, 2). The inflammatory cascade originates in the adventitia and progressively involves all layers of the arterial wall, leading to concentric thickening that may result in structural abnormalities such as stenosis, occlusion, dilatation, or aneurysm formation (3). A well-documented hypercoagulable state in TAK further amplifies the thrombosis risk in vessels with preexisting lesions, often culminating in acute ischemic events (AIE). These events may involve multiple vascular territories, including the cerebral, coronary, mesenteric, renal, and peripheral arteries, and may lead to devastating outcomes such as stroke, myocardial infarction, intestinal ischemia, limb gangrene, and death (4). Notably, AIE may still occur despite immunosuppressive therapy, highlighting the need for a better understanding of the vascular, inflammatory, and immune characteristics accompanying ischemic events in TAK.

The pathogenesis of TAK-associated AIE is complex and multifactorial. Traditional cardiovascular risk factors such as dyslipidemia, fail to fully explain the high thrombosis burden in this population (5), indicating that disease-specific mechanisms play an important role. Cumulative evidences have shown that endothelial dysfunction, systemic inflammation are closely linked to the prothrombotic milieu in TAK (6). Routine inflammatory markers (7–9) including erythrocyte sedimentation rate (ESR), C-reactive protein (CRP) and neutrophil-to-lymphocyte ratio (NLR), are widely used to assess disease activity in clinical practice; however, their relationship with ischemic manifestations in TAK remains incompletely understood. Previous studies have confirmed that plasma cells and serum immunoglobulin levels are associated with disease activity and treatment response (10, 11). As a core proinflammatory cytokine, Interleukin-6 (IL-6) also correlates with inflammatory activity in TAK (12). In addition, T lymphocytes play central roles in granulomatous arterial inflammation (13). Despite these advances, it remains unclear which vascular, inflammatory and immune parameters are independently associated with concurrent AIE in TAK patients.

Several assessment tools have been established for evaluating disease activity in TAK, but each has limitations. The National Institutes of Health criteria relies partly on invasive angiography and have low accuracy for judging pathologically activity (14). The Disease Extent Index for TAK does not integrate acute-phase reactants with imaging findings and demonstrates suboptimal agreement with physician global assessment (15, 16). The widely used Indian Takayasu Clinical Activity Score (ITAS)-2010 and its derivative ITAS-A (17) primarily assess clinical disease activity but do not adequately capture vascular structural damage. Imaging-based scoring systems, such as the PET Vascular Activity Score, provide valuable information regarding vascular inflammation but remain constrained by cost, radiation exposure, and limited accessibility (18). Importantly, these assessment systems were not developed to comprehensively characterize the vascular, inflammatory and immune features associated with AIE, as their primary focus is overall disease activity and vascular progression rather than ischemic manifestations. Although comprehensive vascular imaging is recommended for newly diagnosed TAK patients, occult arterial stenosis and subclinical ischemic lesions may remain undetectable before symptomatic ischemic events occur, indicating that imaging findings alone may not fully reflect the biological characteristics accompanying AIE (19).

Although relevant progress has been made in exploring the pathophysiology and biomarkers of TAK, few studies have systematically evaluated the vascular involvement together with inflammatory and immune parameters in patients with AIE. Therefore, we conducted a retrospective 1:2 matched case-control study to compare vascular characteristics, disease activity, inflammatory markers, immunoglobulins, and lymphocyte subsets between hospitalized TAK patients with and without AIE. We further performed multivariable logistic regression analyses to identify vascular, inflammatory, and immune variables independently associated with concurrent AIE and evaluated the robustness of these cross-sectional associations using prespecified sensitivity analyses. Given the retrospective matched case-control design and the availability of biomarker measurements after or shortly following AIE onset, the present study was intended to characterize exploratory cross-sectional associations rather than to develop a model for prospective prediction of future ischemic events.

## Materials and methods

This retrospective 1:2 matched case-control study was conducted at Shanxi Bethune Hospital, enrolling patients diagnosed with TAK who were admitted between January 2014 and December 2024. All participants met either the 1990 American College of Rheumatology (ACR) or the 2022 ACR/European Alliance of Associations for Rheumatology (EULAR) classification criteria for TAK (20, 21). A key inclusion criterion required all patients to be free from interfering medications for at least 3 months before enrollment, to eliminate drug effects on inflammatory and immune biomarkers. Eligible patients fell into two categories: (1) newly diagnosed, treatment-naive patients without prior use of TAK-related therapies or antithrombotic agents; (2) previously diagnosed patients who had voluntarily or medically discontinued glucocorticoids, immunosuppressants and coagulation-modifying drugs for ≥3 months. Cases included 37 TAK patients who developed AIE during hospitalization. AIE were defined as acute vascular events occurring in the arterial circulation, confirmed by imaging and resulting in tissue infarction or ischemia, attributable to *in situ* arterial thrombosis rather than embolic or atherosclerotic mechanisms. Eligible AIE subtypes consisted of acute ischemic stroke with radiological cerebral infarction, acute myocardial infarction, peripheral arterial thrombosis, and visceral arterial thrombosis. Pulmonary infarction was only included imaging (contrast-enhanced CT, CTA or PET-CT) confirmed pulmonary artery wall thickening, stenosis or occlusion attributable to TAK. Patients with AIE attributable to alternative etiologies were excluded, including: (1) pulmonary infarction secondary to venous thromboembolism without evidence of pulmonary vasculitis; (2) suspected ischemic events without clinical or radiological confirmation; (3) embolic events related to atrial fibrillation, antiphospholipid syndrome or other non-inflammatory conditions; (4) ischemia caused by cardiogenic embolism, non-TAK vascular stenosis or trauma. Controls were 74 TAK patients without AIE, matched to case at a 1:2 ratio by age, sex, and body mass index (BMI). All controls were recruited during the same study period and also satisfied the 3-month medication-free requirement. Additional exclusion criteria for all participants were as follows: (1) coexisting other connective tissue diseases or overlap syndrome; (2) history of malignant tumors, active infections, hematological disorders, severe hepatic or renal insufficiency, pregnancy, or lactation; (3) incomplete medical records; or (4) missing baseline immune and inflammatory biomarker data. This study was conducted in strict compliance with the ethical principles outlined in the Declaration of Helsinki. The study protocol was reviewed and approved by the Ethics Committee of Shanxi Bethune Hospital (Approval No.: LYLL-2025-004/PJ46).

### Specimen collection and laboratory analysis

All clinical data were independently reviewed and extracted by two experienced rheumatologists. Fasting peripheral blood samples were collected on the next morning after hospital admission following a standardized operating protocol. All baseline samples were obtained before initiation of any new TAK-related medications during hospitalization. Emergency anticoagulant treatment for acute AIE was administered after sample collection and thus did not interfere with baseline biomarker results.

Flow cytometry was utilized to quantify the total number of lymphocytes in each of the subpopulations (CD3+ T, CD4+ T, CD8+ T, B and natural killer (NK) cells) isolated from PB samples. Blood cells were collected using a BD FACSCalibur platform (BD Biosciences, Franklin Lakes, NJ, USA) and quantified using MultiSET software (BD Biosciences) after 50 µL of EDTA-anticoagulated venous blood was placed into A and B Trucount tubes (BD Biosciences). The CD3+, CD4+, and CD8+ T cell subsets were identified using the conjugated monoclonal antibodies fluorescein isothiocyanate (FITC)-CD3, allophycocyanin (APC)-CD4, peridinin chlorophyll protein (PerpCP)-CD45, and phycoerythrin (PE)-CD8 ((BD Biosciences). For B and NK cell populations, monoclonal antibodies FITC-CD3, APC-CD19, PerpCP-CD45, and PE-CD16+CD56 were used (BD Biosciences). Subsequently 20 μl of above-mentioned antibodies were added into A and B tubes, respectively, followed by vortex stirring and incubation at room temperature for 15 minutes. Finally, cells were analyzed by flow cytometry and quantified using MultiSET software. Within one hour of collection, blood samples from all TAK patients were centrifuged at 3000g for twenty minutes; the resulting serum was then used for cytokine assays. Using a Cytometric Bead Array, the concentrations of IL-2, IL-4, IL-6, IL-10, IL-17, INF-γ and TNF-α in the serum were determined. Every procedure followed the manufacturer’s instructions.

Disease activity was assessed using the ITAS-2010 (17). The Takayasu damage score (TADS) (22) was applied to evaluate TAK organ damage. Discrepancies between the two reviewers were resolved through consensus discussion. Any disagreements between the two assessors were resolved by group discussion to reach a consensus.

### Statistical analysis

All statistical analyses were performed using SPSS 26.0 (IBM Corp., Armonk, NY, USA) and R software (version 4.5.3; R Foundation for Statistical Computing, Vienna, Austria). A two-sided *p* value <0.05 was considered statistically significant. Continuous variables were assessed for normality using the Shapiro-Wilk test. Normally distributed variables were expressed as mean ± standard deviation (SD) and compared using the independent-samples T test. Non-normally distributed variables were summarized as median (interquartile range, IQR) and compared using the Mann-Whitney U test. Categorical variables were presented as frequencies and percentages and compared using the chi-square test or Fisher’s exact test, as appropriate. Univariate logistic regression analysis was initially performed to identify variables associated with concurrent AIE. Variables with *p*-value < 0.05 in univariate analysis were entered into multivariable logistic regression analyses. Three hierarchical multivariate unconditional logistic regression models were constructed by biological dimension: Model 1 included vascular lesion variables only; Model 2 additionally incorporated inflammatory markers; and Model 3 further included peripheral T-lymphocyte subsets. A prespecified parsimonious sensitivity analysis was performed using biologically relevant vascular, inflammatory, and immune variables to evaluate the robustness of the observed cross-sectional associations and partially address potential model over-parameterization resulting from the limited number of AIE events. Because biomarkers in patients with AIE were measured after or shortly following the ischemic event, all multivariable analyses were intended to identify variables independently associated with concurrent AIE rather than to develop a model for prospective prediction of future ischemic events.

The discriminatory performance of the sequential multivariable association models was evaluated using receiver operating characteristic (ROC) curves and the corresponding area under the curve (AUC).

Internal validation was performed using 1000 bootstrap resamples to assess the stability of model discrimination. The apparent AUC, optimism-corrected AUC, and corresponding 95% confidence intervals (CIs) were calculated, and AUCs were compared using the DeLong test. Calibration curves and the Hosmer-Lemeshow goodness-of-fit test were generated as exploratory within-sample assessments of agreement between fitted probabilities and observed outcomes in this matched case-control dataset. Decision curve analysis (DCA) was likewise performed as an exploratory within-sample analysis to compare the relative statistical performance of the sequential models. Owing to the retrospective matched case-control design and post-event biomarker measurements, the calibration and DCA results were not intended to estimate absolute risk, demonstrate clinical utility, or support clinical decision-making.

## Results

### Clinical features and vascular involvement of TAK patients with and without AIE

**Figure 1:**
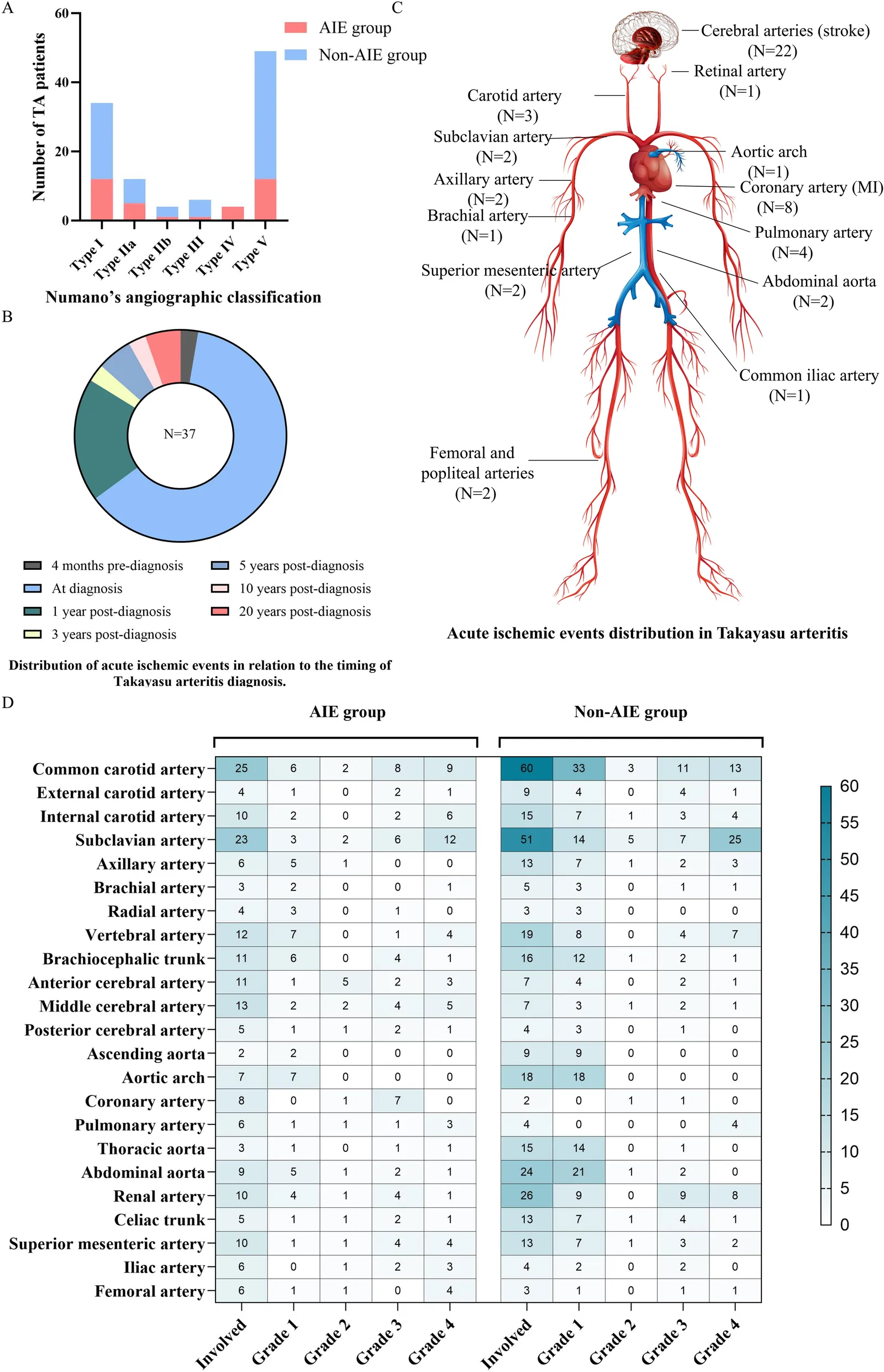
Temporal and anatomical characteristics of arterial thrombotic events in Takayasu arteritis. **(A)** Numano’s angiographic classification of TAK patients with and without AIE. Type I and Type V patterns were more common in the AIE group Note: Two patients with isolated pulmonary artery involvement were not classifiable under the conventional Numano angiographic system and were therefore excluded from **(A)**. **(B)** Temporal distribution of AIE in relation to the time of TAK diagnosis (N = 37). The majority of AIE occurred at diagnosis (62.20%), followed by post-diagnosis intervals at 1 year (18.9%). **(C)** Anatomical distribution of AIE sites in TAK patients. The most frequently affected vascular territory was the cerebral arteries (N = 22), followed by the coronary arteries (N = 8) and pulmonary arteries (N = 4). **(D)** Vascular involvement of TAK patients stratified by AIE status. TAK, Takayasu arteritis; AIE, acute ischemic events; MI, myocardial infarction.

Details of the individual cases are provided in **Supplementary Tables 1**, **2**. A total of 111 patients with TAK were included in this study, comprising 37 patients with AIE and 74 without AIE who served as the control group. The mean age at onset of TAK in the AIE group was 34.92 ± 10.83 years, ranging from 16 to 58 years. No statistically significant differences were found between the AIE and control groups in terms of gender (female: 83.3% *vs* 90.5%, *p* = 0.299) or BMI (22.60 ± 2.76 kg/m2 *vs* 23.18 ± 3.30 kg/m2, *p* = 0.368). The prevalence of smoking or alcohol consumption showed no significant difference between the AIE and control groups. Similarly, no significant differences were observed in the history of hypertension, diabetes or dyslipidemia between the AIE and control groups. The majority of patients with AIE were females, with only 6 male patients, consistent with the known sex distribution of TAK. As classified by Numano’s angiographic criteria (**Figure 1A**), type I and type V were the dominant patterns in the AIE group (12/37, 32.4%). Conversely, type V predominated in patients without AIE (37/74, 50%), followed by type I (22/74, 29.7%). The remaining subtypes were distributed as follows: type IV was observed in 4 patients (4 in the AIE group and 0 in the non-AIE group), type III in 6 (1 and 5, respectively), type IIa in 12 (5 and 7, respectively), and type IIb in 4 (1 and 3, respectively).We further compared vascular involvement between patients with and without AIE (**Figure 1D**, **Supplementary Table 3**). Compared with controls, patients with concurrent AIE exhibited significantly higher frequencies of anterior cerebral artery (ACA) stenosis (*p* = 0.007), middle cerebral artery (MCA) stenosis (*p* = 0.001), coronary artery stenosis (*p* = 0.001) and femoral artery stenosis (*p* = 0.028). No significant differences were observed for the remaining cervicocephalic, aortic, or visceral branch arteries (all *p* > 0.05), although pulmonary and iliac artery involvement showed borderline between-group differences (both *p* = 0.062). The first episode of AIE occurred from the time of initial presentation to over 28 years after the onset of TAK, with a mean duration of TAK of 77.68 ± 108.89 months before AIE onset. The interval between the first disease manifestation and definitive TAK diagnosis averaged 39.14 ± 84.56 months, whereas the mean interval between TAK diagnosis and AIE occurrence was 38.56 ± 72.20 months. Among the 37 patients with AIE, 23 were identified at the time of diagnosis, 1 occurred 4 months before TAK diagnosis, and the rest developed during follow-up at 1 (n=7), 3 (n=1), 5 (n=2), 10 (n=1), and 20 years(n=2) after diagnosis (**Figure 1B**). The anatomical distribution of thrombotic events in TAK is illustrated in **Figure 1C**, with cerebral artery thrombosis (stroke) being the most common ischemic manifestation.

The median follow-up duration was comparable between the two groups (56.64 ± 35.72 *vs* 62.53 ± 55.63 months, *p* = 0.564). Patients with AIE experienced a numerically higher number of disease relapses than controls, although the difference did not reach statistical significance (1.86 ± 2.08 *vs* 1.26 ± 1.60, *p* = 0.091). As summarized in **Supplementary Tables 1**, **2**, severe long-term functional sequelae were substantially more frequent among patients with AIE. Several patients also developed major adverse events (MAEs) during follow-up. Notably, one patient developed pyoderma gangrenosum 14 months after TAK diagnosis and later experienced extensive embolic occlusion involving the anterior and posterior tibial, peroneal, femoral, and popliteal arteries at 50 months post-diagnosis, ultimately requiring a right transfemoral amputation (**Supplementary Figure 1**). This case illustrates the heterogeneous clinical course of TAK and highlights that extensive vascular involvement may occur even several years after diagnosis.

### Inflammation markers, immunity parameters and disease activity in patients with and without concurrent AIE

As summarized in **Table 1**, patients with concurrent AIE exhibited more extensive disease involvement. Seizures were significantly more frequent in the AIE group, and the number of blocked arteries was also significantly higher than that in patients without AIE (both *p* < 0.05). No significant between-group differences were observed in other clinical manifestations, including fever, fatigue, cardiovascular involvement, or other complications.

**Table 1:** The demographic information and clinical features in TAK patients with or without acute ischemic events.

	TAK with AIE (N=37)	TAK without AIE (N=74)	T/Z value	*P* value
Demographic information				
Age, year	34.92±10.83	39.43±12.90	-1.829	0.070
Female,N(%)	31/37(83.3)	67/74(90.5)	-1.039	0.299
BMI, score	22.60±2.76	23.18±3.30	-0.961	0.368
Smoking, N(%)			-0.793	0.429
Never smoked	36/37(97.3)	71/74(95.9)		
Past history of smoking	1/37(2.7)	0/74(0)		
Current smoking	0/37(0)	3/74(4.1)		
History of drinking alcohol, N(%)	0/37(0)	0/74(0)	–	–
Hypertension, N(%)	14/37(37.8)	24/74(32.4)	0.561	0.576
Diabetes mellitus, N(%)	4/37(10.8)	5/74(6.8)	0.733	0.465
Dyslipidemia, N(%)	9/37(24.3)	15/74(20.3)	0.485	0.629
Disease course				
The course of disease, months	77.68±108.89	73.73±95.66	0.196	0.845
Mean time to TAK diagnosis, months	39.14±84.56	35.35±60.86	0.270	0.787
Time from TAK diagnosis to AIE, months	38.56±72.20	–	–	–
New onset of TAK,N(%)	24/37(64.9)	45/74(60.8)	0.412	0.681
ITAS-2010, score	7.46±3.08	5.49±2.56	3.573	**0.001**
Number of involved arteries	7.49±3.44	6.50±3.45	1.423	0.158
Number of blocked arteries	1.89±1.79	1.18±1.47	2.242	**0.027**
Clinical manifestations, N(%)				
Fever	6/37(16.2)	15/74(20.3)	-0.510	0.611
Fatigue	20/37(54.1)	28/74(37.8)	1.631	0.106
Weight loss	0/37(0)	2/74(2.7)	-1.005	0.315
Blurred vision	6/37(16.2)	7/74(9.5)	-1.039	0.299
Amaurosis fugax	3/37(8.1)	7/74(9.5)	-0.232	0.817
Blindness	1/37(2.7)	0/74(0)	-1.414	0.157
Hearing loss	0/37(0)	2/74(2.7)	-1.005	0.315
Syncope	4/37(10.8)	5/74(6.8)	0.733	0.465
Headache	5/37(13.5)	14/74(18.9)	-0.708	0.481
Dizziness	14/37(37.8)	25/74(33.8)	0.418	0.677
Seizure	4/37(10.8)	1/74(1.4)	-2.255	**0.024**
Limb claudication	11/37(29.7)	20/74(27.0)	0.297	0.767
Systolic blood pressure >140 mmHg	15/37(40.5)	20/74(27.0)	-1.438	0.150
Diastolic blood pressure >90 mmHg	12/37(32.4)	14/74(18.9)	-1.578	0.115
Pulse asymmetry	15/37(40.5)	27/74(36.5)	0.412	0.681
Vascular bruit	33/37(89.2)	64/74(86.5)	0.401	0.689
New onset pulselessness	15/37(40.5)	26/74(35.1)	0.552	0.582
Carotidynia	1/37(2.7)	6/74(8.1)	-1.099	0.272
Aortic regurgitation/aortic insufficiency	6/37(16.2)	16/74(21.6)	-0.669	0.505
Sinus tachycardia	3/37(8.1)	3/74(4.1)	0.886	0.378
Pericardial effusion	1/37(2.7)	2/74(2.7)	0.001	1.000
Heart failure	4/37(10.8)	5/74(6.8)	0.733	0.465
Pulmonary hypertension	7/37(18.9)	10/74(13.5)	0.741	0.461
Renal insufficiency	1/37(2.7)	8/74(10.8)	-1.469	0.142
Abdominal pain	5/37(13.5)	4/74(5.4)	-1.469	0.142
Arthralgia and myalgia	2/37(5.4)	9/74(12.2)	-1.118	0.264
Spontaneous abortion	0/37(0)	4/74(5.4)	-1.343	0.152
Aneurysm formation	6/37(16.2)	11/74(14.9)	0.185	0.854
Laboratory index (reference value)				
ESR(mm/h) (0-15)	41.54±35.78	33.75±26.75	1.289	0.200
CRP(mg/L) (0.00-8.00)	19.87±34.04	17.55±28.00	0.384	0.702
NLR	2.79±3.06	2.90±2.00	-0.236	0.814
CHO(mmol/L)(3.0-5.7)	3.77±1.15	4.27±1.31	-1.863	0.066
TG(mmol/L)(0-1.7)	1.38±0.94	1.20±0.77	0.975	0.332
LDL(mmol/L)(0-3.37)	2.30±0.66	2.54±0.86	-1.227	0.223
PT (s)	11.96±0.98	13.42±11.54	-0.752	0.454
FIB-C (g/L)	3.29±.90	3.63±1.22	-1.460	0.147
D-dimer(ng/ml)(0-243)	235.31±519.35	186.86±173.08	0.714	0.477
Homocysteine, μmol/L	24.40±19.54	18.65±12.68	0.989	0.331
C3(g/L)(0.79-1.52)	1.16±0.31	1.19±0.32	-0.375	0.709
C4(g/L)(0.1-0.40)	0.29±0.17	0.28±0.32	0.142	0.887
IgA(g/L)(0.7-4.0)	3.19(2.24,4.98)	2.42(1.76,3.62)	-2.480	**0.013**
IgG(g/L)(7-16)	12.06±5.12	12.18±4.05	-0.138	0.891
IgM(g/L)(0.4-2.3)	1.36±0.57	1.38±0.73	-0.189	0.850
Th cell(cells/μL)(340-1072)	1124.00(851.50,1546.50)	853.50(586.75,1053.00)	-3.954	**0.001**
Ts cell (cells/μL)(210-742)	715.00(507.50,984.50)	487.00(336.50,667.25)	-3.888	**0.001**
B cell (cells/μL)(90-660)	240.97±118.08	241.18±139.90	-0.008	0.994
NK cell (cells/μL)(46-590)	253.68±253.14	210.43±140.99	1.157	0.250
IL-2 (pg/ml) (0-5.71)	0.80(0.43,1.28)	1.08(0.54,2.14)	-1.376	0.169
IL-4 (pg/ml) (0-3.00)	2.46±4.64	2.75±4.21	-0.326	0.745
IL-6 (pg/ml) (0-5.30)	30.50(8.60,49.23)	4.27(2.68,12.12)	-4.413	**0.001**
IL-10 (pg/ml) (0-4.91)	4.12±4.83	4.99±5.89	-0.777	0.439
IL-17 (pg/ml) (0-20.60)	2.79±2.21	3.10±3.27	-0.488	0.626
IFN-γ (pg/ml) (0-7.42)	5.27±9.60	7.53±19.76	-0.656	0.513
TNF-α (pg/ml) (0-4.60)	3.96±7.82	3.30±4.06	0.588	0.558
Follow-up time, months	56.64±35.72	62.53±55.63	-0.579	0.564
Relapses, N	1.86±2.08	1.26±1.60	1.704	0.091
TADS, score	4.00(2.00,5.00)	3.00(2.00,4.00)	-2.043	**0.041**
Death, N(%)	1/37(2.7)	1/74(1.4)	0.501	0.618

TAK: Takayasu arteritis; AIE: acute ischemic events; ESR: erythrocyte sedimentation rate; CRP: C-reactive protein; NLR: neutrophil-to-lymphocyte ratio; CHO: total cholesterol; TG: triglycerides; LDL: low-density lipoprotein cholesterol; PT: prothrombin time; FIB-C: fibrinogen (concentration);C3: Complement 3; C4: Complement 4; IgA: immunoglobulin A; IgG: immunoglobulin G; IgM: immunoglobulin M; Th cell: T helper cell (CD3+CD4+); Ts cell: suppressor T cells (CD3+CD8+); B cell: B cell (CD3-CD19+); NK cell: natural killer cell (CD3-CD56+).

For disease activity and vascular damage, The ITAS-2010 score was markedly elevated in patients with AIE than in controls (7.46 ± 3.08 *vs* 5.49 ± 2.56, *p* < 0.001). The AIE group also exhibited a modestly higher TADS (4.00 [2.00, 5.00] *vs* 3.00 [2.00, 5.00], *p* = 0.041), indicating greater cumulative vascular damage.

Among inflammatory markers, serum IgA levels were significantly higher in the AIE group (3.19 [2.24, 4.98] g/L *vs* 2.42(1.76,3.62) g/L, *p* = 0.013). Serum IL-6 concentrations were also remarkably increased in the AIE group (30.50 [8.60, 49.23] pg/mL *vs* 4.27(2.68,12.12) pg/mL, *p* < 0.001), suggesting a more pronounced inflammatory profile in patients presenting with concurrent AIE.

Analysis of lymphocyte subsets revealed significant between-group differences. Th cell counts were significantly higher in the AIE group (1124.00 [851.50, 1546.50] cells/μL *vs* 853.50[586.75,1053.00] cells/μL, *p*=<0.001). Similarly, Ts cell levels were also markedly elevated in patients with AIE (715.00 [507.50, 984.50] cells/μL *vs* 487.00[336.50,667.25] cells/μL, *p* < 0.001). No significant differences were observed in B cells, NK cells, or other circulating cytokines between the two groups (all *p*>0.05). As shown in **Supplementary Table 4**, autoantibody profiles were comparable between groups.

### Multivariable analysis of factors associated with concurrent AIE in patients with TAK

Based on the aforementioned findings, multivariate logistic regression analyses were performed to identify vascular, inflammatory, and immune variables independently associated with concurrent AIE in TAK, and three sequential hierarchical models were constructed (**Table 2**). Model 1 included vascular involvement only. Coronary artery, MCA and femoral artery were independently associated with concurrent AIE (all *p* < 0.05), whereas ACA stenosis showed a borderline association (*p* = 0.080). The total number of blocked arteries was not independently associated with concurrent AIE (*p* = 0.837). Model 2 incorporated inflammatory variables (ITAS-2010, IgA and IL-6) in addition to vascular lesions. Coronary, ACA, and MCA stenosis remained independently associated with concurrent AIE (all *p* < 0.05), whereas the association for femoral artery stenosis became borderline significant (*p* = 0.064). Higher ITAS-2010 score, serum IgA and IL-6 levels also independently associated with concurrent AIE (all *p* < 0.05). Model 3 further incorporated peripheral T-lymphocyte subsets. In this fully adjusted model, coronary artery stenosis (OR = 18.958, 95% CI: 1.546-232.548, *p* = 0.021), ACA stenosis (OR = 9.450, 95% CI: 1.429-62.480, *p* = 0.020), MCA stenosis (OR = 5.948, 95% CI: 1.179-30.019, *p* = 0.031), femoral artery stenosis (OR = 35.026, 95% CI: 1.409-870.693, *p* = 0.030), higher ITAS-2010 score (OR = 1.327, 95% CI: 1.038-1.696, *p* = 0.024), elevated serum IgA (OR = 1.830, 95% CI: 1.056-3.171, *p* = 0.031), increased IL-6 levels (OR = 1.056, 95% CI: 1.022-1.091, *p* = 0.001), and increased Th cell count (OR = 1.003, 95% CI: 1.001-1.005, *p* = 0.009) remained independently associated with concurrent AIE.

**Table 2:** Multivariate associated factor analysis of TAK patients with acute ischemic events.

Characteristics	Beta	OR	95%(CI)	*P* values
Model 1				
Constant	-1.852			
Number of blocked arteries	0.033	1.034	(0.752,1.421)	0.837
Coronary artery	3.063	21.386	(3.838,119.173)	**0.001**
Anterior cerebral artery	1.184	3.266	(0.868,12.290)	0.080
Middle cerebral artery	1.791	5.993	(1.822,19.708)	**0.003**
Femoral artery	1.993	7.336	(1.381,38.957)	**0.019**
Model 2				
Constant	-6.316			
Number of blocked arteries	-0.261	0.770	(0.511,1.161)	0.212
Coronary artery	2.802	16.476	(2.149,126.298)	**0.007**
Anterior cerebral artery	1.866	6.463	(1.282,32.573)	**0.024**
Middle cerebral artery	1.614	5.021	(1.160,21.725)	**0.031**
Femoral artery	2.084	8.035	(0.889,72.645)	0.064
ITAS-2010	0.305	1.356	(1.092,1.685)	**0.006**
IgA	0.607	1.834	(1.155,2.913)	**0.010**
IL-6	0.046	1.047	(1.020,1.075)	**0.001**
Model 3				
Constant	-10.349			
Number of blocked arteries	0.239	0.787	(0.482,1.285)	0.338
Coronary artery	2.942	18.958	(1.546,232.548)	**0.021**
Anterior cerebral artery	2.246	9.450	(1.429,62.480)	**0.020**
Middle cerebral artery	1.783	5.948	(1.179,30.019)	**0.031**
Femoral artery	3.556	35.026	(1.409,870.693)	**0.030**
ITAS-2010	0.283	1.327	(1.038,1.696)	**0.024**
IgA	0.604	1.830	(1.056,3.171)	**0.031**
IL-6	0.054	1.056	(1.022,1.091)	**0.001**
Th cell	0.003	1.003	(1.001,1.005)	**0.009**
Ts cell	0.001	1.001	(0.998,1.004)	0.510

The Bolded value represents statistical significance.

Model 1 was adjusted according to the number of blocked arteries, coronary artery, anterior cerebral artery, middle cerebral artery and femoral artery; Model 2 was further adjusted for ITAS-2010 score, IgA and IL-6; Model 3 was developed based on Model 2, with adjustments made to account for Th cell and Ts cell.

Full logit regression equations for each model are presented below:

Model 1: Logit(P) = −1.852 + 0.033×Number of blocked arteries + 3.063×Coronary artery + 1.184×Anterior cerebral artery + 1.791×Middle cerebral artery + 1.993×Femoral artery

Model 2: Logit(P) = −6.316 − 0.261×Number of blocked arteries + 2.802×Coronary artery + 1.866×Anterior cerebral artery + 1.614×Middle cerebral artery + 2.084×Femoral artery + 0.305×ITAS-2010 + 0.607×IgA + 0.046×IL-6

Model 3: Logit(P) = −10.349 − 0.239×Number of blocked arteries + 2.942×Coronary artery + 2.246×Anterior cerebral artery + 1.783×Middle cerebral artery + 3.556×Femoral artery + 0.283×ITAS-2010 + 0.604×IgA + 0.054×IL-6 + 0.003×Th cell + 0.001×Ts cell Abbreviations: TAK: Takayasu arteritis; OR: Odds Ratio; CI: Confidence Interval.

To evaluate the robustness of these findings, a prespecified parsimonious sensitivity analysis was performed. The direction and magnitude of the associations remained largely consistent with those observed in the primary model. Although the association for coronary artery stenosis was attenuated and became borderline significant (OR = 6.54, 95% CI: 0.87-48.92, *p* = 0.067), the direction of effect remained unchanged. The associations for MCA stenosis, ITAS-2010 score, serum IgA, IL-6, and Th cell count remained stable across the primary and sensitivity analyses (**Supplementary Table 5**), supporting the robustness of the principal cross-sectional associations.

### Discriminatory performance, internal validation, and exploratory model evaluation

**Figure 2:**
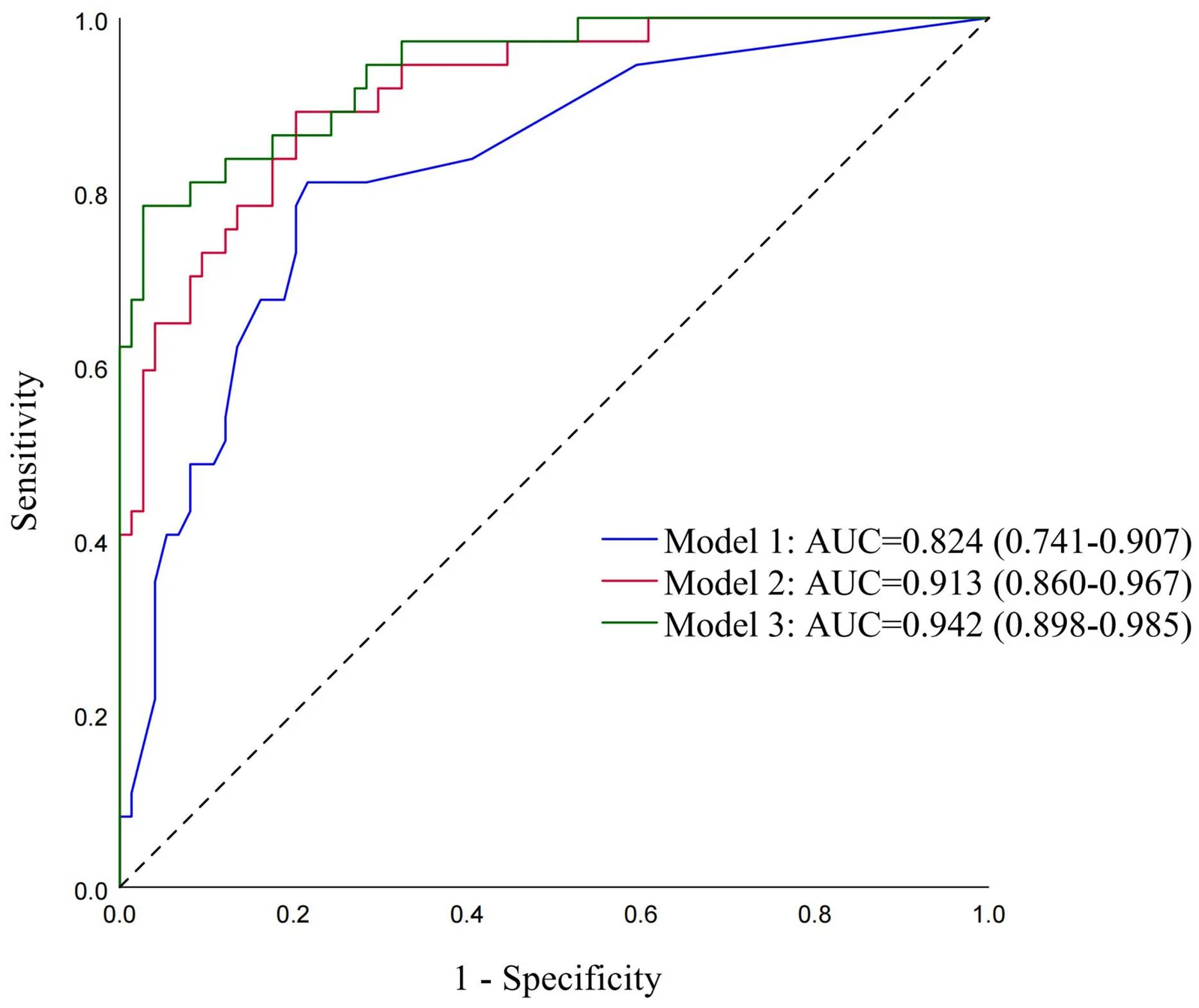
ROC curves of three sequential multivariable association models for concurrent acute ischemic events in Takayasu arteritis. Model 1: Vascular lesion indicators only; Model 2: Vascular lesion indicators plus inflammatory markers; Model 3: Vascular lesion indicators, inflammatory markers, and T lymphocyte subsets.

The discriminatory performance of the three sequential multivariate association regression models was evaluated using ROC curve analysis, 1000-bootstrap internal validation, exploratory calibration curves, and DCA. As illustrated in the ROC plot (**Figure 2**), the apparent AUC values increased progressively across the three models: Model 1 yielded an apparent AUC of 0.824 (95% CI: 0.741-0.907), Model 2 reached 0.913 (95% CI: 0.860-0.967), and the fully adjusted Model 3 attained the highest apparent AUC of 0.942 (95% CI: 0.898-0.985).

After 1000-bootstrap optimism correction, the corrected AUCs remained consistent with the apparent values: 0.794 (95% CI: 0.699-0.880) for Model 1, 0.878 (95% CI: 0.816-0.936) for Model 2, and 0.908 (95% CI: 0.843-0.959) for Model 3. Similar improvements were observed for the Brier score. The apparent Brier scores were 0.1548, 0.1104, and 0.0846 for Model 1-3, respectively, whereas the optimism-corrected Brier scores were 0.1773, 0.1423, and 0.1191, indicating improved within-sample discrimination and overall statistical fit with the sequential incorporation of inflammatory and immune variables. Pairwise comparisons of AUCs using the DeLong test demonstrated significant differences between Model 1 and Model 2 (AUC difference = -0.090, z = -2.645, *p* = 0.008), as well as between Model 1 and Model 3 (AUC difference = -0.118, z = -3.432, *p* = 0.001). Although the AUC difference between Model 2 and Model 3 did not reach statistical significance (AUC difference = -0.028, z = -1.770, *p* = 0.077), a likelihood ratio test demonstrated that adding Th and Ts cell counts significantly improved model fit (χ²=15.86, df = 2, *p* < 0.001).

**Figure 3:**
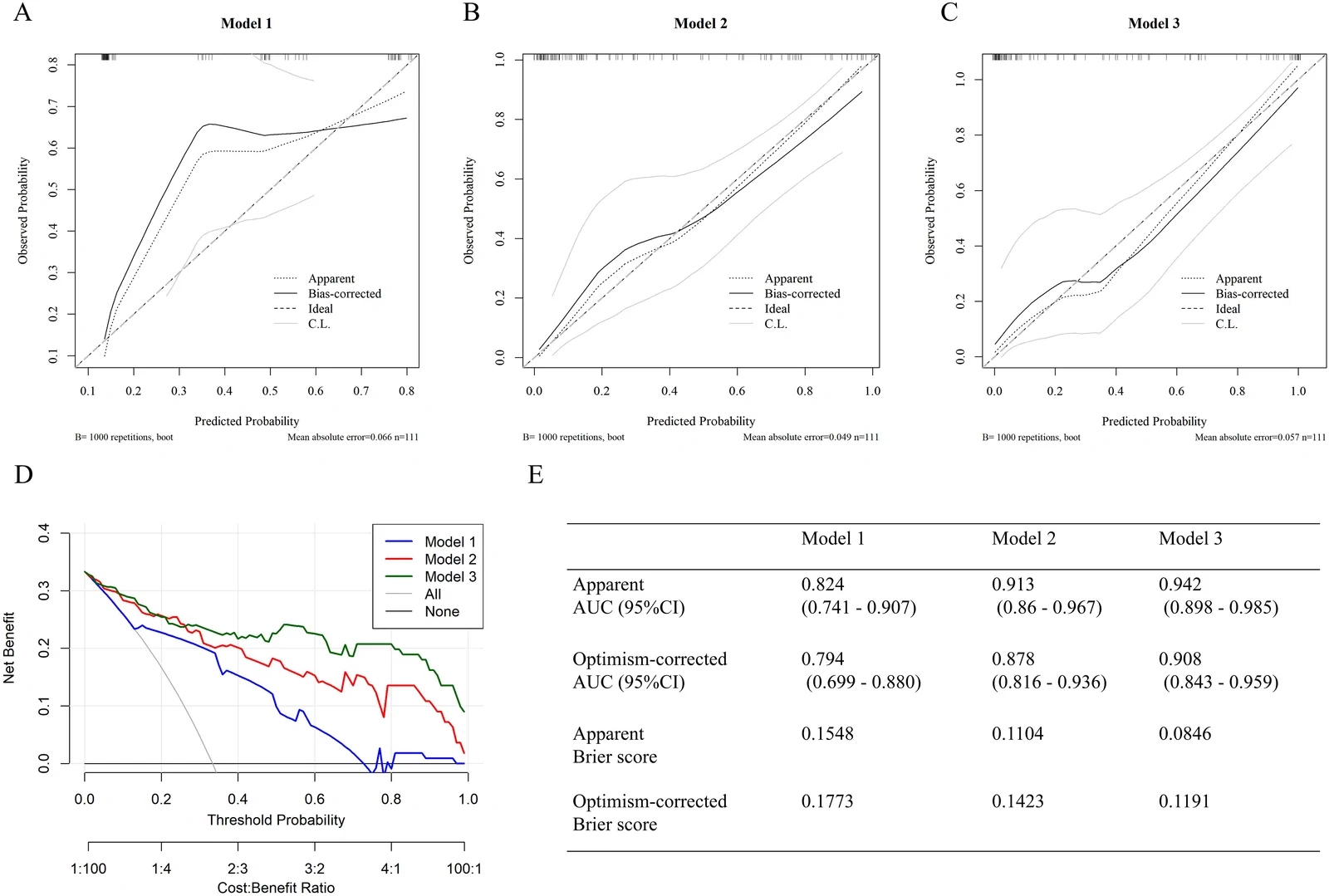
Bootstrap internal validation and within-sample performance assessment of the three sequential multivariable association models. **(A-C)** Bootstrap-calibrated calibration curves (1000 resamples) for Model 1-3. Dotted = apparent curve, solid black = bias-corrected curve, dashed = ideal calibration, light gray=95% confidence limits. Mean absolute calibration error is labeled per panel. Calibration curves are presented as exploratory within-sample assessments. **(D)** Decision curve analysis comparing the relative within-sample net benefit of the three sequential association models across threshold probabilities. Because of the matched case-control design, these curves are presented for exploratory statistical comparison and should not be interpreted as evidence of prospective clinical utility. Gray line = treat-all strategy; black horizontal line = treat-none strategy. **(E)** Summary of apparent and optimism-corrected AUC (95% CI) and Brier scores for the three sequential association models. Model 1: Vascular lesion indicators only; Model 2: Vascular lesion indicators plus inflammatory markers; Model 3: Vascular lesion indicators, inflammatory markers, and T lymphocyte subsets.

Calibration analysis (**Figures 3A–C**) was performed as an exploratory within-sample assessment of agreement between fitted probabilities and observed outcomes in this matched case-control dataset. The mean absolute calibration error was 0.066 for Model 1, 0.049 for Model 2, 0.057 for Model 3. Among the three models, Model 3 demonstrated the closest agreement between fitted probabilities and observed outcomes, with its bias-corrected calibration curve closely approximating the ideal reference line and the 95% confidence band encompassing the reference diagonal. Decision curve analysis (**Figure 3D**) was likewise performed as an exploratory within-sample comparison of the relative statistical performance of the three sequential models. Owing to the retrospective matched case-control design and the availability of biomarker measurements after or shortly following AIE onset, these analyses were not intended to estimate absolute risk, demonstrate clinical utility, or support prospective clinical decision-making. Across threshold probabilities of approximately 0.05-0.80, Model 3 exhibited the highest net benefit among the three models, whereas the net benefit of all models gradually declined at threshold probabilities exceeding 0.80. Overall, these findings indicate that integrating vascular, inflammatory, and immune variables improved the within-sample discriminatory performance of the association models; however, these results should be interpreted as exploratory statistical summaries rather than evidence supporting prospective risk stratification or prediction of future AIE.

## Discussion

AIE represent one of the most devastating complications of TAK and substantially contribute to long-term disability and mortality. In this 10-year single-center retrospective matched case-control study, we systematically evaluated vascular, inflammatory, and immune characteristics in patients with and without concurrent AIE. We found that stenosis involving the coronary arteries, ACA, MCA, and femoral arteries, together with higher ITAS-2010 scores, elevated serum IgA and IL-6 levels, and increased peripheral Th cell counts, were independently associated with concurrent AIE after multivariable adjustment. Furthermore, sequential multivariable association models suggested that integrating vascular, inflammatory, and immune variables provided a more comprehensive characterization of the biological profile observed in patients presenting with AIE than vascular features alone. These findings extend previous observations regarding the immune-inflammatory basis of ischemic complications in TAK. Accordingly, these findings should be regarded as exploratory associations that warrant confirmation in independent prospective cohorts.

Multiple ethnic cohort studies (23–26) have consistently demonstrated that ischemic complications in TAK are closely related to the extent and distribution of vascular involvement. In the present study, Numano type I and type V angiographic patterns predominated among patients with AIE, whereas isolated type V disease was most common in patients without AIE. More importantly, stenosis involving ACA, MCA, coronary artery, and femoral artery remained independently associated with AIE after multivariable adjustment, whereas the total number of occluded arteries showed no independent association. These findings suggest that the anatomical distribution of vascular lesions may be more informative than overall vascular burden in characterizing ischemic complications in TAK. A previous meta-analysis (27) reported that stroke and myocardial infarction occur in approximately 8.9% and 3.4% of patients with TAK, respectively, underscoring the substantial burden of ischemic complications in this disease. The predominance of cerebrovascular events in our cohort is consistent with these observations and is biologically plausible. Consistent with our findings, a 320-patient TAK cohort reported cerebrovascular ischemia occurred in 20% of patients, frequently accompanied by multivessel stenosis (26). Similarly, a long-term survival study including 224 patients identified stroke and myocardial infarction as the leading cause of death in TAK, with mortality substantially exceeding that of the age- and sex-matched general populations (SMR = 17.29) (4). Femoral artery involvement may reflect extensive systemic vasculitis rather than isolated peripheral arterial disease. Supporting this interpretation, an FDG-PET/CT study of 77 patients found that femoral artery inflammation was associated with older age, male sex, and a higher burden of atherosclerotic risk factors (28).

Patients with AIE exhibited higher ITAS-2010 scores, more severe vascular occlusion, and higher TADS, supporting that sustained vascular inflammation and cumulative structural damage are closely linked to ischemic complications in TAK (29). Conventional inflammatory markers (ESR, CRP and NLR) showed no independent correlation with AIE, consistent with prior work indicating these routine indicators poorly reflect long-term thrombotic predisposition (7–9). We also evaluated traditional cardiovascular risk factors potentially related to arterial thrombosis. Although serum homocysteine levels were slightly elevated in the AIE group, no significant intergroup difference emerged, consistent with previous studies (30, 31). Collectively, these findings suggest that conventional cardiovascular risk factors and routinely used inflammatory biomarkers have limited ability to capture the heterogeneous immune-inflammatory status accompanying ischemic complications in TAK. In contrast, serum IgA and IL-6 levels were significantly higher in patients with AIE and remained independently associated with AIE after multivariable adjustment. Rather than reflecting nonspecific systemic inflammation alone, these biomarkers may better characterize the immune-inflammatory milieu accompanying ischemic events in TAK. Their independent associations observed in the present study further support the concept that immune activation represents an important component of the biological characteristics of patients presenting with AIE and provides a rationale for investigating these pathways in future mechanistic and prospective studies.

The vascular inflammation characteristic of TAK is driven by complex interactions between humoral and cellular immunity. In the present study, serum IgA, IL-6, and peripheral Th cell counts were all independently associated with concurrent AIE after multivariable adjustment, supporting the involvement of adaptive immune activation in patients presenting with ischemic complications. Although the cross-sectional design precludes causal inference, these findings extend previous observations by demonstrating that both humoral and cellular immune responses are closely linked to the immune-inflammatory characteristics accompanying AIE. Serum IgA was significantly elevated in patients with AIE and was verified as an independent AIE-associated factor after adjusting for confounding variables. Previous studies have suggested that increased IgA may reflect persistent mucosal or systemic immune activation, and IgA-mediated immune complexes can promote endothelial injury, platelet aggregation, and arterial thrombosis (11, 32, 33). Our findings expand the potential clinical relevance of IgA in TAK by suggesting that, beyond its reported association with disease activity, elevated IgA also characterizes patients presenting with ischemic complications. IL-6 is a pivotal cytokine in the inflammatory network of TAK and has consistently been associated with disease activity and vascular inflammation. In our cohort, IL-6 showed a prominent correlation with AIE and was markedly higher in patients with ischemic complications. Cumulative preclinical and clinical evidence (12, 34) confirms excessive IL-6 signaling promotes endothelial dysfunction, enhances acute-phase responses, and disrupt the balance between coagulation and fibrinolysis, thereby contributing to a prothrombotic vascular environment. The present findings further support the close relationship between IL-6-mediated immune activation and ischemic manifestations in TAK. Abnormal T-cell activation represents another hallmark of TAK pathogenesis (35). Peripheral Th cell counts were independently associated with AIE, whereas Ts cell counts were no longer significant after adjustment for other variables. CD4+ Th cells are central mediators of granulomatous arterial inflammation and contribute to vascular remodeling through the production of pro-inflammatory cytokines, including IL-6 and IFN-γ (36, 37). Moreover, activated T lymphocytes participate in the crosstalk between inflammation and coagulation within the injured vascular wall (13). Although the present findings cannot establish temporal or causal relationships, they support the concept that adaptive immune activation constitutes an important biological feature of TAK patients presenting with concurrent AIE.

To better evaluate the relative contributions of vascular, inflammatory, and immune variables, we constructed three sequential multivariate association models. The stepwise incorporation of inflammatory biomarkers and T-lymphocyte subsets progressively improved within-sample discrimination, while likelihood ratio testing demonstrated that the addition of T-cell subsets significantly improved overall model fit. Although the difference in AUC between Models 2 and 3 did not reach statistical significance, the bootstrap-corrected discrimination remained stable, suggesting that immune variables contributed additional information beyond vascular and inflammatory characteristics alone. Given the relatively limited number of AIE events, we further performed a prespecified parsimonious sensitivity analysis to address potential model over-parameterization. The principal associations for MCA stenosis, ITAS-2010 score, serum IgA, IL-6, and Th cell count remained largely unchanged after model simplification. Although the association for coronary artery stenosis became borderline significant in the sensitivity analysis, the direction and magnitude of the association were generally preserved, suggesting that the principal findings were reasonably robust despite the limited sample size. Nevertheless, the wide confidence intervals observed for several vascular variables indicate limited precision of the regression estimates and should be interpreted with appropriate caution. To provide a comprehensive statistical evaluation, we additionally performed bootstrap internal validation, calibration analysis, and DCA. These analyses consistently supported the overall statistical performance of the sequential association models within the study sample. However, because this was a retrospective matched case-control study with biomarker measurements obtained after or shortly following AIE onset, the calibration and decision curve analyses should be regarded as exploratory assessments of within-sample model performance rather than evidence of prospective prediction, absolute risk estimation, or clinical decision-making utility. External validation in independent prospective multicenter cohorts remains essential before any broader application of these statistical models can be considered.

The present findings provide additional insight into the vascular, inflammatory, and immune characteristics of TAK patients presenting with concurrent AIE. Rather than relying on conventional inflammatory markers alone, integrating information on vascular involvement, disease activity, humoral immunity, and T-cell activation may offer a more comprehensive understanding of the biological heterogeneity accompanying ischemic complications in TAK. These observations complement previous studies focusing predominantly on angiographic manifestations by highlighting the potential contribution of adaptive immune activation to ischemic phenotypes. From a clinical perspective, the identified vascular and immune-inflammatory variables may assist in the comprehensive assessment of patients presenting with AIE, although they should not be interpreted as validated predictors of future ischemic events. Instead, these findings generate hypotheses for future mechanistic investigations and prospective longitudinal studies designed to clarify the temporal relationship between immune activation and ischemic complications. Such studies will be essential to determine whether dynamic changes in these biomarkers precede AIE onset and whether they provide incremental value beyond conventional clinical and imaging assessments.

Several limitations of this study should be acknowledged. First, this was a retrospective single-center matched case-control study, and fasting blood samples in patients with AIE were obtained at admission, mostly after or shortly following the ischemic event. Therefore, the temporal relationship between immune biomarkers and AIE cannot be established, and reverse causation cannot be excluded. Second, the enriched case-control design restricted to treatment-naïve patients without recent glucocorticoid, immunosuppressive, or antithrombotic therapy may have introduced selection bias and limits the generalizability of our findings to broader TAK populations. Third, the relatively small number of AIE events limited statistical power and increased the risk of model over-parameterization, as reflected by the wide confidence intervals for several vascular variables. Although a prespecified parsimonious sensitivity analysis demonstrated generally consistent associations, larger prospective cohorts using prespecified or penalized regression approaches are needed to confirm these findings. Fourth, the included ischemic events represented a heterogeneous group of clinical outcomes, and no prespecified prediction horizon was defined. Consequently, the multivariable models should be interpreted as exploratory analyses of cross-sectional associations rather than tools for prospective risk prediction. Finally, only internal bootstrap validation was performed, peripheral immune biomarkers were measured at a single time point, and detailed T-cell phenotyping was unavailable. External multicenter validation with longitudinal serial immune profiling will be essential to verify these observations and further clarify the mechanisms underlying ischemic complications in TAK.

In conclusion, patients with TAK presenting with AIE exhibited higher disease activity, more extensive vascular involvement, and enhanced humoral and cellular immune activation. Stenosis of the coronary, anterior cerebral, middle cerebral, and femoral arteries, together with higher ITAS-2010 scores, elevated serum IgA and IL-6 levels, and increased peripheral Th cell counts, were independently associated with concurrent AIE. These findings expand current understanding of the vascular and immune-inflammatory characteristics associated with ischemic complications in TAK and provide a foundation for future prospective studies to determine their temporal and prognostic significance.

## Data Availability

The datasets presented in this study can be found in online repositories. The names of the repository/repositories and accession number(s) can be found in the article/**Supplementary Material**.

## Supplementary material

Supplementary Figure 1 Progressive pyoderma gangrenosum and thrombotic arterial occlusion in a Takayasu arteritis patient with major adverse vascular outcome. **(A–H)** show the cutaneous course of pyoderma gangrenosum in a 51-year-old male patient, beginning 14 months after the diagnosis of TAK. The lesion initially presented as a painful necrotic nodule **(A)**, progressively enlarged with central ulceration and violaceous borders **(B–E)**, and developed into widespread ulceration and hemorrhagic necrosis **(F, G)**. Panel H shows the healed skin 3 years later with post-inflammatory scarring and atrophy following immunosuppressive therapy. **(I, J)** show CT angiography images acquired 50 months after TAK diagnosis, revealing extensive embolic occlusion involving the anterior and posterior tibial, peroneal, femoral, and popliteal arteries. Progressive limb ischemia led to right transfemoral amputation. TAK, Takayasu arteritis.

Supplementary Table 1 Detailed clinical profiles of the 37 TAK patients with acute ischemic events.

Supplementary Table 2 Major adverse events and/or sequelae in 74 TAK patients without acute ischemic events.

Supplementary Table 3 Vascular involvement of TAK patients stratified by AIE status.

Supplementary Table 4 The laboratory features of the TAK patients with or without acute ischemic events.

Supplementary Table 5 Sensitivity analysis using a prespecified parsimonious multivariable logistic regression model.

## References

[1] Mruthyunjaya P Misra R . Update on takayasu arteritis: Year in review 2024. Int J Rheum Dis. (2024) 27:e15314. doi: 10.1111/1756-185X.15314 39221891

[2] Kermani TA Tomelleri A Scaramuzzi D Jagtap S Misra DP . Management of takayasu arteritis - a 2026 update. Curr Rheumatol Rep. (2026) 28:8. doi: 10.1007/s11926-026-01214-2 41860699

[3] Arnaud L Haroche J Mathian A Gorochov G Amoura Z . Pathogenesis of takayasu's arteritis: a 2011 update. Autoimmun Rev. (2011) 11:61–7. doi: 10.1016/j.autrev.2011.08.001 21855656

[4] Jagtap S Mishra P Rathore U Thakare DR Singh K Dixit J . Increased mortality rate in takayasu arteritis is largely driven by cardiovascular disease: a cohort study. Rheumatol (Oxford). (2024) 63:3337–45. doi: 10.1093/rheumatology/kead584 37934123

[5] Zang Q Li F Ju Y Wang J Luo J Liu W . Dysregulated serum lipid profile and atherosclerosis in untreated female takayasu arteritis patients: a propensity score-matched analysis. Scand J Rheumatol. (2025) 54:302–10. doi: 10.1080/03009742.2025.2488096 40326057

[6] Aksu K Donmez A Keser G . Inflammation-induced thrombosis: mechanisms, disease associations and management. Curr Pharm Des. (2012) 18:1478–93. doi: 10.2174/138161212799504731 22364132

[7] Sun Y Cui X Kong X Chen H Wu S Ma L . The role of plateletcrit in takayasu arteritis: a potential biomarker for disease activity and 6-month treatment response. Int J Rheum Dis. (2023) 26:2517–25. doi: 10.1111/1756-185X.14951 37875306

[8] Pathadan AP Tyagi S Gupta MD M PG Mahajan S Kunal S . The study of novel inflammatory markers in takayasu arteritis and its correlation with disease activity. Indian Heart J. (2021) 73:640–3. doi: 10.1016/j.ihj.2021.08.002 34627584 PMC8514415

[9] Seringec Akkececi N Yildirim Cetin G Gogebakan H Acipayam C . The c-reactive protein/albumin ratio and complete blood count parameters as indicators of disease activity in patients with takayasu arteritis. Med Sci Monit. (2019) 25:1401–9. doi: 10.12659/MSM.912495 30792377 PMC6396438

[10] Guo Y Du J Li T Gao N Yang S Zhang Y . Elevated serum immunoglobulin level predicts high risk of 1-year recurrence in patients with takayasu arteritis. Arthritis Res Ther. (2023) 25:36. doi: 10.1186/s13075-023-03016-8 36882846 PMC9990310

[11] Vantaggio L Pellicano C Miglionico M Cusano G Visentini M . Might iga be a biomarker of disease activity in takayasu arteritis? Eur J Case Rep Intern Med. (2022) 9:3664. doi: 10.12890/2022_003664 36632539 PMC9829015

[12] Souza Pedreira AL Leite de Castro Flores M Barreto Santiago M . Interleukin 6 levels and disease activity in takayasu arteritis: a systematic review with meta-analysis. J Clin Rheumatol. (2024) 30:58–64. doi: 10.1097/RHU.0000000000002053 38190729

[13] Saadoun D Garrido M Comarmond C Desbois AC Domont F Savey L . Th1 and th17 cytokines drive inflammation in takayasu arteritis. Arthritis Rheumatol. (2015) 67:1353–60. doi: 10.1002/art.39037 25604824

[14] Misra DP Jain N Ora M Singh K Agarwal V Sharma A . Outcome measures and biomarkers for disease assessment in takayasu arteritis. Diagnostics (Basel). (2022) 12(10):2565. doi: 10.3390/diagnostics12102565 36292253 PMC9601573

[15] Boleto G Berti A Merkel PA Aydin SZ Direskeneli H Dejaco C . Measurement properties of outcome instruments for large-vessel vasculitis: a systematic literature review. J Rheumatol. (2023) 50:789–98. doi: 10.3899/jrheum.220149 36319004

[16] Alibaz-Oner F Aydin SZ Akar S Aksu K Kamali S Yucel E . Assessment of patients with takayasu arteritis in routine practice with Indian takayasu clinical activity score. J Rheumatol. (2015) 42:1443–7. doi: 10.3899/jrheum.140817 26136490

[17] Misra R Danda D Rajappa SM Ghosh A Gupta R Mahendranath KM . Development and initial validation of the Indian takayasu clinical activity score (ITAS2010). Rheumatol (Oxford). (2013) 52:1795–801. doi: 10.1093/rheumatology/ket128 23594468

[18] Katakura T Shirai T . Positron emission tomography in takayasu arteritis: a review including patterns of vascular involvement across modalities and regions. J Clin Med. (2025) 14(9):2939. doi: 10.3390/jcm14092939 40363971 PMC12073023

[19] Soto ME Melendez-Ramirez G Herrera Zarza MC Meave A Cruz Marmolejo MA Mendez-Dominguez A . Comprehensive magnetic resonance imaging evaluation in a cohort of 145 patients with takayasu's arteritis. Proposal for a standardised and systematic reporting format. Clin Exp Rheumatol. (2026) 44:695–707. doi: 10.55563/clinexprheumatol/4bmcp0 41105443

[20] Arend WP Michel BA Bloch DA Hunder GG Calabrese LH Edworthy SM . The american college of rheumatology 1990 criteria for the classification of takayasu arteritis. Arthritis Rheum. (1990) 33:1129–34. doi: 10.1002/art.1780330811 1975175

[21] Grayson PC Ponte C Suppiah R Robson JC Gribbons KB Judge A . 2022 american college of rheumatology/EULAR classification criteria for takayasu arteritis. Ann Rheum Dis. (2022) 81:1654–60. doi: 10.1136/ard-2022-223482 36351705

[22] Exley AR Bacon PA Luqmani RA Kitas GD Gordon C Savage CO . Development and initial validation of the vasculitis damage index for the standardized clinical assessment of damage in the systemic vasculitides. Arthritis Rheum. (1997) 40:371–80. doi: 10.1002/art.1780400222 9041949

[23] Bicakcigil M Aksu K Kamali S Ozbalkan Z Ates A Karadag O . Takayasu's arteritis in Turkey - clinical and angiographic features of 248 patients. Clin Exp Rheumatol. (2009) 27:S59-64. 19646348

[24] Ahn SS Han M Park YB Jung I Lee SW . Incidence, prevalence and risk of stroke in patients with takayasu arteritis: a nationwide population-based study in South Korea. Stroke Vasc Neurol. (2022) 7:149–57. doi: 10.1136/svn-2020-000809 34880114 PMC9067261

[25] Kong F Huang X Su L Liao Q Wang C Zhao Y . Risk factors for cerebral infarction in takayasu arteritis: a single-centre case-control study. Rheumatol (Oxford). (2021) 61:281–90. doi: 10.1093/rheumatology/keab308 33774663

[26] Mirouse A Deltour S Leclercq D Squara PA Pouchelon C Comarmond C . Cerebrovascular ischemic events in patients with takayasu arteritis. Stroke. (2022) 53:1550–7. doi: 10.1161/STROKEAHA.121.034445 35354303

[27] Kim H Barra L . Ischemic complications in takayasu's arteritis: a meta-analysis. Semin Arthritis Rheum. (2018) 47:900–6. doi: 10.1016/j.semarthrit.2017.11.001 29198409

[28] Kaymaz-Tahra S Ozguven S Avcu A Filizoglu N Unal AU Ones T . Involvement of iliofemoral arteries in PET/CT is associated with atherosclerotic risk factors in takayasu's arteritis. J Clin Med. (2025) 14(23):8607. doi: 10.3390/jcm14238607 41375908 PMC12692745

[29] Immanuel J Yun S . Vascular inflammatory diseases and endothelial phenotypes. Cells. (2023) 12(12):1640. doi: 10.3390/cells12121640 37371110 PMC10297687

[30] Marzioglu Ozdemir E Ozdemir G . Inflammation and thrombophilia markers in supra-aortic takayasu arteritis-associated stroke: a digital subtraction angiography-based case control study. J Clin Med. (2026) 15(6):2308. doi: 10.3390/jcm15062308 41899232 PMC13026892

[31] Jin X Yang Y Wang X Wang C Zhao Y . An update on stroke and transient ischemic attack in takayasu arteritis: a systematic review and meta-analysis. Joint Bone Spine. (2026) 93:105951. doi: 10.1016/j.jbspin.2025.105951 40712738

[32] Chen XQ Tu L Tang Q Huang L Qin YH . An emerging role for neutrophil extracellular traps in iga vasculitis: a mini-review. Front Immunol. (2022) 13:912929. doi: 10.3389/fimmu.2022.912929 35799774 PMC9253285

[33] Xu L Li Y Wu X . Iga vasculitis update: epidemiology, pathogenesis, and biomarkers. Front Immunol. (2022) 13:921864. doi: 10.3389/fimmu.2022.921864 36263029 PMC9574357

[34] Webb CE Vautrinot J Hers I . IL-6 as a mediator of platelet hyper-responsiveness. Cells. (2025) 14(11):766. doi: 10.3390/cells14110766 40497942 PMC12153796

[35] Misra DP Singh K Sharma A Agarwal V . Arterial wall fibrosis in takayasu arteritis and its potential for therapeutic modulation. Front Immunol. (2023) 14:1174249. doi: 10.3389/fimmu.2023.1174249 37256147 PMC10225504

[36] Bhandari S Butt SRR Ishfaq A Attaallah MH Ekhator C Halappa Nagaraj R . Pathophysiology, diagnosis, and management of takayasu arteritis: a review of current advances. Cureus. (2023) 15:e42667. doi: 10.7759/cureus.42667 37525862 PMC10386905

[37] Gao N Tang H Li T Yang Y Zhao H Wang L . Single-cell transcriptome analysis reveals cellular heterogeneity in the aortas of takayasu arteritis. Arthritis Res Ther. (2025) 27:55. doi: 10.1186/s13075-025-03523-w 40065428 PMC11892157

