# Supplementary material for: Vascular, inflammatory and immune factors associated with acute ischemic events in Takayasu arteritis: a 10-year retrospective matched case-control study
Source: Front Immunol. 2026 Sep 11;17:1866057. doi: 10.3389/fimmu.2026.1866057 (PMC13612344; doi:10.3389/fimmu.2026.1866057)
Supplement: Supplementary file 1 [file Table1.docx]

Supplement Table 1：Detailed clinical profiles of the 37 TAK patients with acute ischemic events.

| Patient No. | Sex | Age  (years) | Relapse count | Acute ischemic events | Time of AIE relative to TAK diagnosis | Affected vessels | Imaging modality | Exclusion of other embolic sources | Time of biomarker sampling relative to AIE onset | MAEs/Sequelae description | Survive status |
| --- | --- | --- | --- | --- | --- | --- | --- | --- | --- | --- | --- |
| 1. | Male | 44 | 8 | Acute infarction of the left frontal lobe | 28y post-dx | Left middle cerebral artery frontal branch | Brain MRI-DWI+cerebral CTA+cervical CTA | Echo (-); no carotid atherosclerosis; no DVT | After event | Persistent limb claudication, intermittent limb numbness and pain | Alive |
| 2. | Female | 29 | 1 | Right frontal/periventricular/basal ganglia infarction + hemorrhagic infarction secondary to large cerebral artery thrombosis | at dx | Right internal carotid artery territory | Brain MRI-DWI+MRA+cerebral CTA+cervical CTA | Echo (-); no carotid plaque stenosis; no DVT | After event | Left complete hemiplegia, grade 0 limb muscle strength | Alive |
| 3. | Male | 51 | 2 | Bilateral lower-limb limb artery occlusion (femoral/popliteal/tibial/peroneal) due to TAK arterial mural thrombosis | 50mo post-dx | Femoral, popliteal, anterior/posterior tibial, peroneal arteries | CTA of the lower abdomen, pelvis and lower extremity | Echo (-); no atherosclerotic peripheral stenosis; no DVT | After event | Pyoderma gangrenosum (14mo post-dx); right transfemoral amputation | Alive |
| 4. | Female | 23 | 0 | Occlusion of the anterior cerebral artery with infarctions in the left basal ganglia, periventricular region, frontal lobe, and insular cortex | at dx | Left anterior cerebral artery | Brain MRI-DWI+cerebral CTA | Echo (-); no extracranial atherosclerotic stenosis;no DVT | After event | Mild right-sided limb weakness | Alive |
| 5. | Female | 25 | 1 | Temporo-occipital lobe infarction from intracranial arterial stenosis/thrombosis | 5mo post-dx | Left posterior cerebral artery territory | Brain MRI-DWI+MRA+cerebral CTA+cervical CTA | Echo (-); no carotid atherosclerosis; no DVT | After event | Left-sided limb numbness and left visual field defect | Alive |
| 6. | Female | 55 | 0 | Left parietal and basal ganglia cerebral infarction from intracranial large-vessel thrombosis | 13y post-dx | Left intracranial internal carotid artery branches | Brain MRI-DWI+MRA+cerebral CTA+cervical CTA+chest CTA | Echo (-); no carotid atherosclerosis; no DVT | After event | Subclavian steal syndrome and dizziness | Alive |
| 7. | Male | 19 | 0 | Acute myocardial infarction+Superior mesenteric artery mural thrombosis with acute intestinal ischemia | 1mo post-dx | Coronary arteries, superior mesenteric artery origin | Abdominal CTA+coronary CTA | No coronary atherosclerotic stenosis; no cardioembolic source | After event | Intermittent dizziness | Alive |
| 8. | Female | 40 | 4 | Acute infarctions in the left frontal and temporal lobes, and bilateral parietal lobes+ left retinal artery occlusion from intracranial arterial thrombosis | at dx | Bilateral MCA, left ophthalmic artery | Brain MRI-DWI+MRA+fundus angiography+cerebral CTA+cervical CTA | Echo (-); no carotid atherosclerosis; no DVT | After event | Permanent blindness, limb weakness, dysarthria, chronic headache and dizziness | Alive |
| 9. | Female | 30 | 0 | Acute infarctions in the left insular cortex, basal ganglia, periventricular region, and frontoparietal lobes | at dx | Left MCA, insular and periventricular branches | Brain MRI-DWI+MRA | Echo (-); no carotid atherosclerosis; no DVT | After event | Secondary intracerebral hemorrhage (1mo post-dx); limb weakness, syncope, recurrent seizures | Alive |
| 10. | Female | 27 | 1 | Bilateral frontal and right parietal subacute infarction secondary to cerebral large-vessel thrombosis | at dx | Bilateral anterior cerebral artery branches | Brain MRI-DWI+MRA+CTA of the chest, abdomen and pelvis | Echo (-); no carotid atherosclerosis; no DVT | After event | Infarction-induced hypotension, coma, chronic dizziness | Alive |
| 11. | Female | 58 | 2 | Medulla oblongata and pontine infarction from vertebrobasilar artery thrombosis | 5y / 5y6mo post-dx | Bilateral vertebral-basilar artery system | Brain MRI-DWI+MRA | Echo (-); no carotid atherosclerosis; no DVT | After event | Hemiplegia | Alive |
| 12. | Female | 38 | 0 | Acute myocardial infarction+Abdominal aortic dissection false lumen mural thrombosis with acute visceral ischemia | at dx | Coronary arteries, abdominal aorta false lumen branches | Coronary angiography+CTA of the chest, abdomen and pelvis | Coronary arteries without atherosclerotic plaque; no DVT | After event | Abdominal pain | Alive |
| 13. | Female | 34 | 1 | Left frontal and basal ganglia acute cerebral infarction from intracranial arterial occlusion | at dx | Left internal carotid artery branches | Brain MRI-DWI+cerebral CTA+cervical CTA | Echo (-); no carotid atherosclerosis; no DVT | After event | Descending aorta mural thrombus + pulmonary infarction (11y post-dx); right hemiplegia with numbness | Alive |
| 14. | Female | 31 | 2 | Right frontal/parietal/centrum semiovale infarction from MCA thrombosis | at dx | Right middle cerebral artery | Brain MRI-DWI+MRA+cerebral CTA+cervical CTA | Echo (-); no carotid atherosclerosis; no DVT | After event | Recurrent right frontal lobe infarction (2y post-dx); permanent left hemiplegia | Alive |
| 15. | Female | 28 | 1 | Multiple acute cerebral infarctions with hemorrhagic transformation in left frontal, parietal, insular lobes, basal ganglia, and periventricular area | 8mo post-dx | Left MCA, insular and periventricular territories | Brain MRI-DWI+MRA+cerebral CTA+cervical CTA | Echo (-); no carotid atherosclerosis; no DVT | After event | Visual blurring, dizziness, right limb weakness, aphasia | Alive |
| 16. | Female | 43 | 1 | Large cerebral infarction with hemorrhagic transformation in right temporal and parietal lobes | 1y post-dx | Right MCA territory | Brain MRI-DWI+ MRA | Echo (-); no carotid atherosclerosis; no DVT | After event | Left limb weakness, dizziness | Alive |
| 17. | Female | 30 | 2 | Acute infarcts in left basal ganglia, periventricular area, frontal, temporal, and insular cortices | at dx | Left internal carotid and MCA branches | Brain MRI-DWI+MRA | Echo (-); no carotid atherosclerosis; no DVT | After event | Dizziness, transient visual blackout | Alive |
| 18. | Female | 28 | 1 | Acute infarcts in left caudate head and cortex at diagnosis | at dx | Left lenticulostriate arteries | Brain MRI-DWI+MRA | Echo (-); no carotid atherosclerosis; no DVT | After event | Dizziness, dysarthria, right limb weakness | Alive |
| 19. | Female | 32 | 2 | Pulmonary arteries embolism | 3m post-dx | Left pulmonary artery | CTPA | Echo (-); no carotid atherosclerosis; no DVT | After event | Back pain | Alive |
| 20. | Male | 29 | 3 | Cerebral infarctions in the right periventricular region, basal ganglia, and left frontal lobe | at dx | Bilateral MCA and periventricular branches | Brain MRI-DWI+cerebral CTA+cervical CTA | Echo (-); no carotid atherosclerosis; no DVT | After event | Recurrent periventricular infarction (5y/6y post-dx); hemiplegia, mobility impairment, dysarthria | Alive |
| 21. | Male | 56 | 1 | Left common iliac artery aneurysm with thromboembolism | at dx | Left common iliac artery | CTA of the chest, abdomen and pelvis | Coronary arteries normal; no atherosclerotic peripheral disease; no DVT | After event | Abdominal aortic aneurysm impending rupture (16mo post-dx); intermittent claudication | Alive |
| 22. | Female | 27 | 4 | Acute infarctions in the left precentral gyrus, centrum semiovale, periventricular region, basal ganglia, and insular cortex | at dx | Left MCA and periventricular branches | Brain MRI-DWI+cerebral CTA+cervical CTA | Echo (-); no carotid atherosclerosis; no DVT | After event | Recurrent identical territorial infarction (1y post-dx); dysarthria, right facial droop, upper limb paralysis, seizure disorder | Alive |
| 23. | Female | 33 | 0 | Left basal ganglia infarction from intracranial arterial stenosis | 4mo pre-dx | Left lenticulostriate arteries | Brain MRI-DWI+cerebral CTA+cervical CTA | No cardiogenic embolic source; no DVT | After event | Right-sided limb weakness | Alive |
| 24. | Female | 52 | 4 | Right subclavian artery acute thrombosis with upper limb ischemia | at dx | Right subclavian artery arch | CTA of upper extremities, the chest, abdomen and pelvis+cerebral CTA+cervical CTA | No cardiogenic embolic source; no DVT | After event | Right femoral vein recanalized thrombosis (19y post-dx); dizziness | Alive |
| 25. | Female | 34 | 3 | Right pulmonary artery intraluminal thrombosis with pulmonary parenchymal ischemia | at dx | Right middle/lower lobe pulmonary arteries | CTPA | No intracardiac thrombus; no DVT | After event | Hemoptysis | Alive |
| 26. | Female | 47 | 8 | Acute non-ST-elevation myocardial infarction | 8y post-dx | Coronary arteries | Coronary angiography | Coronary arteries without atherosclerotic plaque; no DVT | After event | Fatigue | Alive |
| 27. | Female | 20 | 0 | True aneurysms with thrombosis in the right carotid and right axillary arteries | at dx | Right carotid, right axillary artery | CTA of upper extremities, the chest, abdomen and pelvis+cerebral CTA+cervical CTA | No embolic source detected | After event | none | Alive |
| 28. | Female | 46 | 5 | Bilateral basal ganglia lacunar infarctions from intracranial small-vessel thrombosis | at dx | Bilateral lenticulostriate arteries | Brain MRI-DWI+MRA | Echo (-); no carotid atherosclerosis; no DVT | After event | Dysarthria, severe pulmonary hypertension (81 mmHg), and dizziness | Alive |
| 29. | Male | 16 | 2 | Left cerebral arterial infarction with secondary subdural hematoma | 8mo post-dx | Left MCA territory | Brain MRI-DWI+cerebral CTA+cervical CTA | No intracardiac thrombus; no DVT | After event | - | Dead |
| 30. | Female | 36 | 2 | Acute myocardial infarction+Carotid artery mural thrombosis | 3y post-dx | Coronary arteries; right common carotid artery | Coronary angiogram+cervical ultrasound + cervical CTA | No coronary atherosclerotic stenosis; no DVT | After event | Dizziness and transient vision loss | Alive |
| 31. | Female | 22 | 1 | Left subclavian-axillary artery long-segment thrombosis with upper limb ischemia | 6mo post-dx | Left subclavian to axillary artery | CTA of upper extremities, the chest, abdomen and pelvis+cerebral CTA+cervical CTA | Echo (-); no peripheral atherosclerosis; no DVT | After event | Dizziness | Alive |
| 32. | Female | 27 | 0 | Acute myocardial infarction, thrombosis of the right common carotid artery | at dx | Coronary arteries; right common carotid artery | Coronary angiogram+cerebral CTA+cervical CTA | No cardiac embolic source; no peripheral atherosclerosis; no DVT | After event | None | Alive |
| 33. | Female | 33 | 0 | Thrombosis of the middle cerebral artery with focal infarction in the left frontal lobe | at dx | Left middle cerebral artery | Brain MRI-DWI+cerebral MRA | Echo (-); no peripheral atherosclerosis; no DVT | After event | Hemiplegia with limited mobility | Alive |
| 34. | Female | 39 | 0 | Acute myocardial infarction, pulmonary artery thrombosis, and brachial artery thrombosis | at dx | Coronary arteries; pulmonary artery; bilateral brachial, | Coronary angiogram+CTA of upper extremities+cervical CTA+CTPA | No cardiogenic embolic source; no peripheral atherosclerosis; no DVT | After event | None | Alive |
| 35. | Female | 34 | 2 | Acute myocardial infarction, superior mesenteric artery branch thrombosis with intestinal ischemia | at dx | Coronary arteries; superior mesenteric artery branches | Coronary angiogram+CTA of chest, abdomen and pelvis | No intracardiac thrombus; no peripheral atherosclerosis; no DVT | After event | Jejunal vein thrombosis (8mo pre-dx, non-qualifying); small bowel resection; recurrent abdominal pain | Alive |
| 36. | Female | 30 | 0 | Acute cerebral infarctions in left basal ganglia, left occipital lobe, and bilateral frontal lobes | at dx | Left MCA, occipital and frontal branches | Brain MRI-DWI+cerebral MRA | Echo (-); no peripheral atherosclerosis; no DVT | After event | Mild limb weakness | Alive |
| 37. | Male | 46 | 5 | Acute myocardial infarction, abdominal aortic thrombosis | at dx | Coronary arteries; abdominal artery | Coronary angiogram+CTA of chest, abdomen and pelvis | Echo (-); no peripheral atherosclerosis; no DVT | After event | Recurrent heart failure | Alive |

Time intervals for events are reported relative to TAK diagnosis.

TAK: Takayasu arteritis; Dx:diagnosis; MAEs: major adverse events; MCA: middle cerebral artery；MRI : magnetic resonance imaging; DWI: diffusion-weighted imaging; MRA: magnetic resonance angiography; CTA: computed tomography angiography; CTPA: CT pulmonary angiography; DVT: deep vein thrombosis.
