# Supplementary material for: Vascular, inflammatory and immune factors associated with acute ischemic events in Takayasu arteritis: a 10-year retrospective matched case-control study
Source: Front Immunol. 2026 Sep 11;17:1866057. doi: 10.3389/fimmu.2026.1866057 (PMC13612344; doi:10.3389/fimmu.2026.1866057)
Supplement: Supplementary file 2 [file Table2.docx]

Supplement Table 2：Major adverse events and/or sequelae in 74 TAK patients without acute ischemic events.

| Patient No. | Sex | Age  (years) | Relapse count | MAEs/Sequelae description | Survive status |
| --- | --- | --- | --- | --- | --- |
| 38. | Female | 27 | 0 | None | Alive |
| 39. | Female | 32 | 0 | Ascending aortic aneurysm-like dilation without impact on daily life. | Alive |
| 40. | Male | 60 | 0 | None | Alive |
| 41. | Female | 49 | 0 | Intermittent joint pain | Alive |
| 42. | Female | 41 | 2 | Femoral head necrosis (2y post-dx, corticosteroid-related); femoral neck fracture; heart failure,. | Alive |
| 43. | Female | 43 | 7 | Abdominal aortic ulcer, osteoporosis, recurrent in-stent restenosis | Alive |
| 44 | Female | 17 | 0 | Impaired right upper limb mobility and finger dexterity, with difficulty writing and using chopsticks | Alive |
| 45. | Female | 42 | 0 | None | Alive |
| 46. | Female | 42 | 0 | Dizziness, limb weakness, transient binocular blindness, upper limb numbness, limping | Alive |
| 47. | Female | 44 | 1 | Dizziness, bilateral upper limb weakness, limping, transient binocular blindness, blurred vision | Alive |
| 48. | Female | 50 | 0 | Left upper limb weakness, limping, left renal atrophy, exertional dyspnea, heart failure | Alive |
| 49. | Male | 55 | 3 | Abdominal pain | Alive |
| 50. | Female | 31 | 1 | Left upper arm soreness and fatigue, limping | Alive |
| 51. | Male | 61 | 2 | Limb fatigue and numbness | Alive |
| 52. | Female | 72 | 1 | Bilateral upper limb weakness | Alive |
| 53. | Female | 37 | 1 | Heart failure | Alive |
| 54. | Female | 26 | 1 | Hypertension | Alive |
| 55. | Female | 55 | 0 | Hypertension | Alive |
| 56. | Male | 24 | 0 | Right renal atrophy | Alive |
| 57. | Female | 39 | 0 | Dizziness, left limb numbness | Alive |
| 58. | Female | 25 | 0 | Headache | Alive |
| 59. | Female | 45 | 1 | Dizziness with blurred vision | Alive |
| 60. | Female | 37 | 6 | Chest and back pain | Alive |
| 61. | Female | 21 | 0 | Dizziness | Alive |
| 62. | Female | 50 | 3 | Interstitial fibrosis, pulselessness | Alive |
| 63. | Female | 32 | 1 | Chest and back pain | Alive |
| 64. | Female | 53 | 4 | Left parietal lobe hemorrhage (1 mo pre-dx); intracerebral hemorrhage with pneumonia and respiratory failure (2 y post-dx); limb dysfunction, dysphasia, hemiplegia, epilepsy | Alive |
| 65. | Female | 30 | 1 | Left upper limb weakness | Alive |
| 66. | Female | 30 | 0 | Widened pulse pressure | Alive |
| 67. | Female | 42 | 0 | Dizziness | Alive |
| 68. | Female | 43 | 0 | Lower limb claudication, bilateral upper limb weakness | Alive |
| 69. | Female | 63 | 0 | Left upper limb fatigue and weakness | Alive |
| 70. | Female | 58 | 0 | Pulselessness | Alive |
| 71. | Female | 40 | 1 | Left upper arm soreness and weakness | Alive |
| 72. | Female | 26 | 0 | Widened pulse pressure | Alive |
| 73. | Female | 38 | 1 | Chest and back pain | Alive |
| 74. | Female | 28 | 0 | None | Alive |
| 75. | Male | 66 | 4 | Heart failure, limping, upper limb numbness | Alive |
| 76. | Female | 27 | 1 | Dilated cardiomyopathy, pulmonary hypertension, significant exertional dyspnea, and reduced tolerance to daily activities | Alive |
| 77. | Female | 38 | 0 | Dizziness | Alive |
| 78. | Male | 37 | 0 | Headache | Alive |
| 79. | Female | 50 | 0 | Headache | Alive |
| 80. | Female | 46 | 0 | Dizziness, hypertrophic cardiomyopathy, subclavian steal syndrome | Alive |
| 81. | Female | 39 | 0 | None | Alive |
| 82. | Female | 23 | 2 | Pyoderma gangrenosum (5 years post-dx); pulmonary tuberculosis (6 years post-dx); dizziness, fatigue, pyoderma gangrenosum | Alive |
| 83. | Female | 34 | 0 | None | Alive |
| 84. | Female | 50 | 0 | Fatigue | Alive |
| 85. | Female | 36 | 2 | Neck and back pain, headache | Alive |
| 86. | Female | 37 | 1 | None | Alive |
| 87. | Female | 51 | 4 | Limping | Alive |
| 88. | Female | 60 | 2 | Osteonecrosis (5 y post-dx, corticosteroid-related);  interstitial lung disease (2 y post-dx); generalized pain, Dyspnea | Alive |
| 89. | Female | 34 | 0 | Headache, hypertension | Alive |
| 90. | Female | 27 | 1 | Transient vision blackout | Alive |
| 91. | Female | 29 | 0 | Limping, dizziness | Alive |
| 92. | Male | 57 | 0 | Limping, dizziness | Alive |
| 93. | Female | 56 | 0 | Dizziness | Alive |
| 94. | Female | 28 | 1 | Dizziness | Alive |
| 95. | Female | 34 | 3 | Headache, limb weakness | Alive |
| 96. | Female | 44 | 2 | Limping, dizziness | Alive |
| 97. | Female | 42 | 0 | Dizziness | Alive |
| 98. | Female | 50 | 3 | Dizziness | Alive |
| 99. | Female | 27 | 6 | Neck pain | Alive |
| 100. | Female | 24 | 2 | Left basal ganglia hemorrhage with coma (50 mo post-dx) | Dead |
| 101. | Female | 22 | 1 | None | Alive |
| 102. | Female | 32 | 4 | Chest tightness, dyspnea, heart failure | Alive |
| 103. | Female | 61 | 3 | Dizziness, sudden right-sided hearing loss | Alive |
| 104. | Female | 18 | 3 | Fatigue | Alive |
| 105. | Female | 29 | 1 | Headache, hypertension | Alive |
| 106. | Female | 49 | 1 | Fatigue | Alive |
| 107. | Female | 24 | 1 | Shoulder and back pain | Alive |
| 108. | Female | 20 | 2 | None | Alive |
| 109. | Female | 32 | 2 | Hypertension | Alive |
| 110. | Female | 48 | 3 | Dizziness and headache | Alive |
| 111. | Female | 29 | 1 | Headache | Alive |

TAK: Takayasu arteritis; Dx:diagnosis; MAEs: major adverse events.
