# Supplementary material for: Vascular, inflammatory and immune factors associated with acute ischemic events in Takayasu arteritis: a 10-year retrospective matched case-control study
Source: Front Immunol. 2026 Sep 11;17:1866057. doi: 10.3389/fimmu.2026.1866057 (PMC13612344; doi:10.3389/fimmu.2026.1866057)
Supplement: Supplementary file 3 [file Table3.docx]

Supplement Table 3：Vascular involvement of TAK patients stratified by AIE status.

| Vascular involvement | TAK with AIE  (N=37) | TAK without AIE  (N=74) | T/Z value | *P* value |
| --- | --- | --- | --- | --- |
| Common carotid artery | 25 | 60 | -1.578 | 0.115 |
| External carotid artery | 4 | 9 | -0.207 | 0.836 |
| Internal carotid artery | 10 | 15 | 0.798 | 0.426 |
| Subclavian artery | 23 | 51 | -0.707 | 0.481 |
| Axillary artery | 6 | 13 | -0.177 | 0.860 |
| Brachial artery | 3 | 5 | 0.257 | 0.797 |
| Radial artery | 4 | 3 | -1.374 | 0.169 |
| Vertebral artery | 12 | 19 | 0.743 | 0.459 |
| Brachiocephalic trunk | 11 | 16 | 0.934 | 0.352 |
| Anterior cerebral artery | 11 | 7 | -2.719 | **0.007** |
| Middle cerebral artery | 13 | 7 | -3.303 | **0.001** |
| Posterior cerebral artery | 5 | 4 | -1.469 | 0.142 |
| Ascending aorta | 2 | 9 | -1.118 | 0.264 |
| Aortic arch | 7 | 18 | -0.640 | 0.522 |
| Coronary artery | 8 | 2 | -3.267 | **0.001** |
| Pulmonary artery | 6 | 4 | -1.867 | 0.062 |
| Thoracic aorta | 3 | 15 | -1.631 | 0.103 |
| Abdominal aorta | 9 | 24 | -0.876 | 0.383 |
| Renal artery | 10 | 26 | -0.855 | 0.394 |
| Celiac trunk | 5 | 13 | -0.542 | 0.589 |
| Superior mesenteric artery | 10 | 13 | -1.154 | 0.249 |
| Iliac artery | 6 | 4 | -1.867 | 0.062 |
| Femoral artery | 6 | 3 | -2.203 | **0.028** |

TAK: Takayasu arteritis; AIE: acute ischemic events.
