# Supplementary material for: Vascular, inflammatory and immune factors associated with acute ischemic events in Takayasu arteritis: a 10-year retrospective matched case-control study
Source: Front Immunol. 2026 Sep 11;17:1866057. doi: 10.3389/fimmu.2026.1866057 (PMC13612344; doi:10.3389/fimmu.2026.1866057)
Supplement: Supplementary file 4 [file Table4.docx]

Supplement Table 4: The laboratory features of the TAK patients with or without acute ischemic events.

| Laboratory index  (reference value) | TAK with AIE  (N=37) | TAK without AIE  (N=74) | T/Z  value | *P*  value |
| --- | --- | --- | --- | --- |
| WBC (×10^9^/L) (3.5-9.5) | 7.86±2.79 | 7.30±2.72 | 1.012 | 0.314 |
| N (×10^9^/L) (1.80-6.3) | 4.96±2.98 | 4.81±2.31 | 0.297 | 0.767 |
| HGB(g/L) (115-150) | 123.05±18.22 | 115.23±22.99 | 1.803 | 0.074 |
| LY(×10^9^/L) (1.10-3.20) | 2.08±0.56 | 1.84±0.62 | 1.945 | 0.054 |
| PLT(×10^9^/L) (125-350) | 282.07±99.80 | 278.18±94.15 | 0.201 | 0.841 |
| ALT(IU/L)(7-40) | 20.40(9.90,37.55) | 16.85(11.33,22.70) | -1.298 | 0.194 |
| AST(IU/L)(13-35) | 20.00(14.55,28.50) | 16.55(13.95,21.30) | -1.811 | 0.070 |
| ALP(IU/L)(35-100) | 68.05(55.50,79.90) | 66.40(53.68,94.78) | -0.914 | 0.361 |
| GGT(IU/L)(7-45) | 23.99±12.60 | 27.86±23.01 | -0.934 | 0.352 |
| ALB(g/L)(40-55) | 38.71±4.04 | 37.70±4.31 | 1.156 | 0.250 |
| Cr(umol/L)(50-96) | 63.95±13.57 | 72.91±35.85 | -1.146 | 0.146 |
| BUN(mmol/L)(2.6-7.5) | 4.86±1.57 | 4.53±1.58 | 1.036 | 0.302 |
| hs-cTnI(pg/ml)(0-11.6) | 6.70(0.02,65.00) | 1.80(0.03,6.80) | -1.080 | 0.280 |
| BNP(pg/ml)(0-100) | 316.40±452.38 | 306.00±493.14 | 0.054 | 0.957 |
| 25 hydroxyvitamin D | 15.18±6.67 | 16.80±2.32 | -0.631 | 0.535 |
| TSH(μIU/ml)(0.56-5.91) | 2.37±2.49 | 2.56±1.76 | -0.380 | 0.705 |
| FT3(pg/ml)(2.30-4.80) | 2.80±0.64 | 3.23±1.85 | -1.211 | 0.230 |
| FT4(ng/dl)(0.59-1.25) | 0.99±0.46 | 1.00±0.54 | -0.194 | 0.847 |
| Autoantibodies (N/%) |  |  |  |  |
| ANA | 10/37(27.0) | 25/74(33.8) | -0.717 | 0.475 |
| ACL(GPL/MPL) | 2/37(5.4) | 5/74(6.8) | -0.274 | 0.785 |
| ACL-IgG | 2/37(5.4) | 3/74(4.1) | 0.321 | 0.749 |
| ACL-IgM | 0/37(0) | 2/74(2.7) | -1.005 | 0.315 |
| ACL-IgA | 1/37(2.7) | 0/74(0) | -1.414 | 0.157 |
| Anti β2GPI(GPL/MPL) | 1/37(2.7) | 1/72(1.4) | 0.501 | 0.618 |
| Anti β2GPI-IgG | 0/37(0) | 1/72(1.4) | -0.705 | 0.482 |
| Anti β2GPI-IgM | 1/37(2.7) | 0/74(0) | -1.414 | 0.157 |
| Anti β2GPI-IgA | 0/37(0) | 0/74(0) | - | - |
| LA | 2/37(5.4) | 1/72(1.4) | -1.236 | 0.216 |
| P-ANCA | 1/37(2.7) | 2/74(2.7) | 0.001 | 1.000 |
| C-ANCA | 0/37(0) | 0/74(0) | - | - |
| Anti-MPO antibody | 0/37(0) | 0/74(0) | - | - |
| Anti-PR3 antibody | 0/37(0) | 0/74(0) | - | - |
| Decreased protein C | 0/37(0) | 0/74(0) | - | - |
| Decreased protein S | 0/37(0) | 4/74(5.4) | -1.434 | 0.152 |

TAK: Takayasu arteritis; AIE: acute ischemic events; WBC: total white blood cell; N: absolute neutrophil count; HGB: hemoglobin; LY: absolute lymphocyte count; PLT: Total platelet counts; ALT: alanine aminotransferase; AST: aspartate aminotransferase; ALP: alkaline phosphatase; GGT:gamma-glutamyltransferase; ALB: albumin; Cr: creatinine; BUN: blood urea nitrogen; hs-cTnI: High-sensitivity cardiac troponin I; BNP: brain natriuretic peptide; TSH: thyroid stimulating hormone; Anti-dsDNA: anti-double-stranded DNA, AHA: anti-histone antibody; AnuA: anti-nucleosome antibody; ACL: anti-cardiolipin antibody; Anti-β2-GPI: Anti-β2-glycoprotein I antibody; LA: lupus anticoagulant. P-ANCA: perinuclear anti-neutrophil cytoplasmic antibody; C-ANCA: cytoplasmic anti-Neutrophil cytoplasmic antibody; MPO: myeloperoxidase; PR3: proteinase 3.
