# Supplementary material for: Vascular, inflammatory and immune factors associated with acute ischemic events in Takayasu arteritis: a 10-year retrospective matched case-control study
Source: Front Immunol. 2026 Sep 11;17:1866057. doi: 10.3389/fimmu.2026.1866057 (PMC13612344; doi:10.3389/fimmu.2026.1866057)
Supplement: Supplementary file 5 [file Table5.docx]

Supplement Table 5: Sensitivity analysis using a prespecified parsimonious multivariable logistic regression model

| **Variable** | Primary model (Model 3) | | | Parsimonious sensitivity model | | |
| --- | --- | --- | --- | --- | --- | --- |
|  | OR | 95%(CI) | *P* values | OR | 95%(CI) | *P* values |
| Coronary artery | 18.958 | (1.546,232.548) | **0.021** | 6.538 | (0.874,48.921) | **0.067** |
| Middle cerebral artery | 5.948 | (1.179,30.019) | **0.031** | 8.407 | (2.000,35.339) | **0.004** |
| ITAS-2010 | 1.327 | (1.038,1.696) | **0.024** | 1.293 | (1.045,1.600) | **0.018** |
| IgA | 1.830 | (1.056,3.171) | **0.031** | 1.818 | (1.103,2.996) | **0.019** |
| IL-6 | 1.056 | (1.022,1.091) | **0.001** | 1.052 | (1.022,1.084) | **0.001** |
| Th cell | 1.003 | (1.001,1.005) | **0.009** | 1.003 | (1.001,1.004) | **0.001** |

Note: Sensitivity model included coronary artery stenosis, middle cerebral artery stenosis, ITAS-2010, serum IgA, IL-6, and Th cell count. Variables were prespecified according to biological plausibility and the primary multivariable analysis rather than selected solely on statistical significance. The sensitivity analysis was performed to evaluate the robustness of the observed cross-sectional associations given the limited number of AIE events.
